# ParABS System in Chromosome Partitioning in the Bacterium *Myxococcus xanthus*

**DOI:** 10.1371/journal.pone.0086897

**Published:** 2014-01-22

**Authors:** Antonio A. Iniesta

**Affiliations:** Departamento de Genética y Microbiología, Área de Genética, Facultad de Biología, Universidad de Murcia, Campus Regional de Excelencia Internacional “Campus Mare Nostrum”, Murcia, Spain; Loyola University Medical Center, United States of America

## Abstract

Chromosome segregation is an essential cellular function in eukaryotic and prokaryotic cells. The ParABS system is a fundamental player for a mitosis-like process in chromosome partitioning in many bacterial species. This work shows that the social bacterium *Myxococcus xanthus* also uses the ParABS system for chromosome segregation. Its large prokaryotic genome of 9.1 Mb contains 22 *parS* sequences near the origin of replication, and it is shown here that *M. xanthus* ParB binds preferentially to a consensus *parS* sequence *in vitro*. ParB and ParA are essential for cell viability in *M. xanthus* as in *Caulobacter crescentus*, but unlike in many other bacteria. Absence of ParB results in anucleate cells, chromosome segregation defects and loss of viability. Analysis of ParA subcellular localization shows that it clusters at the poles in all cells, and in some, in the DNA-free cell division plane between two chromosomal DNA masses. This ParA localization pattern depends on ParB but not on FtsZ. ParB inhibits the nonspecific interaction of ParA with DNA, and ParA colocalizes with chromosomal DNA only when ParB is depleted. The subcellular localization of ParB suggests a single ParB-*parS* complex localized at the edge of the nucleoid, next to a polar ParA cluster, with a second ParB-*parS* complex migrating after the replication of *parS* takes place to the opposite nucleoid edge, next to the other polar ParA cluster.

## Introduction

Genetic information is *written* in long DNA molecules. A typical bacterial chromosome extends to a length over a thousand times greater than the cell in which it resides. Therefore, chromosomal DNA organization, its transcription, replication, and segregation must be highly organized in the cytoplasm and tightly coordinated in time [1], [2]. Negative DNA supercoiling is the main mechanism for bacterial chromosome compaction, generating topological domains of about 10 kb, the interwound DNA loops and their boundaries being highly dynamic [3]. In *Escherichia coli*, a higher-order structure of chromosomal DNA (or macrodomain) has been described, with a length between 0.8 to 1 Mb, which is organized by MatP protein and multiple *matS* DNA sequences [4]. In *Bacillus subtilis*, the nucleoid adopts an organization where the origins of chromosomal replication (*oriC*) are located near opposite cell poles and termini (*ter*) at the mid-cell [5]. In *Pseudomonas aeruginosa*, the *oriC-ter* axis is oriented from the old pole of the cell to the cell division plane or to the incipient newborn pole [6]. And in a newly divided *Caulobacter crescentus* cell, loci occupy specific regions in the cytoplasmic space with respect to its linear genomic position, being *oriC* at the old cell pole and *ter* at the newborn pole [7]. Unlike eukaryotic cells, chromosome segregation is coupled to chromosome replication, and loci separate progressively just after being replicated [7]–[9]. Models for chromosome segregation without mitotic-like apparatus have been proposed [10]–[13]. However, it is assumed that a chromosomal ParABS system, originally described in plasmids, acts as a mitotic-like apparatus to segregate replicated chromosomes [1], [2], [14]. ParABS systems, which have been identified in over two hundred bacterial chromosomes, consist of three components. One is the *cis*-acting *parS* site that is highly conserved among bacterial species, and is located in the *oriC*-proximal region of the chromosome. The majority of bacterial species have between one and four repeats of putative *parS* sites, although several with five to eight repeats, and even a few with twenty or more, are known [15]. The second component of ParABS systems, protein ParB, binds to the *parS* sites to form a large nucleoprotein complex near *oriC* as well as to the third component of the system, ParA. The latter is an ATPase proposed as the element that provides the force for the segregation of the “centromeric” *parS* sites via dynamic polymerization-depolymerization events [2], [14], [16]–[18]. Genes encoding *parA* and *parB* also are usually found in the *oriC*-proximal regions of the chromosome, and they have been shown to participate in proper chromosome partitioning in numerous bacteria [15], [19].

*Myxococcus xanthus* is a Gram-negative soil δ-proteobacterium used as a prokaryotic model for the investigation of several processes involved in multicellular development, coordinated cell movements, and cellular responses to external signals such as light [20]–[23]. *M. xanthus* has a single large circular chromosome of about 9.14 Mbp. It has been suggested that this enlarged genome (and those of related myxobacteria of the order myxococcales), is a consequence of extensive, but not random, gene duplications whose subsequent divergence enabled evolution of the signaling systems required for the striking multicellular lifestyle of myxobacteria [24]. For all these reasons, it is of particular interest to study the organization of the chromosome and its segregation in this bacterium. The main objective of this work was to ascertain if the DNA sequences that encode the predicted ParABS elements, taken from the *M. xanthus* genome annotation, have a role in chromosome segregation in *M. xanthus*. A second objective was to determine if these ParABS elements are physically or functionally interconnected. A conclusion of this work is that ParB binds preferentially to a *parS* consensus sequence *in vitro*. It is also shown here that ParB is essential for viability and its absence generates anucleate cells demonstrating the key role of ParB in chromosome segregation. ParA is also essential for viability and localizes in DNA-free zones such as at the cell poles and along the cell division plane, prior to cell division. However, this localization pattern is independent of FtsZ. ParA subcellular positioning depends on ParB, the absence of which causes ParA to be delocalized from DNA-free zones and colocalizes with chromosomal DNA. Therefore, ParB appears to prevent colocalization of ParA and DNA by guiding ParA to DNA-free zones. In most cells, ParB localizes at the edge of the chromosomal DNA in subpolar positions to thereby limit ParA localization to polar clusters.

## Results and Discussion

### *parABS* Loci in *M. xanthus*

**Figure 1:**
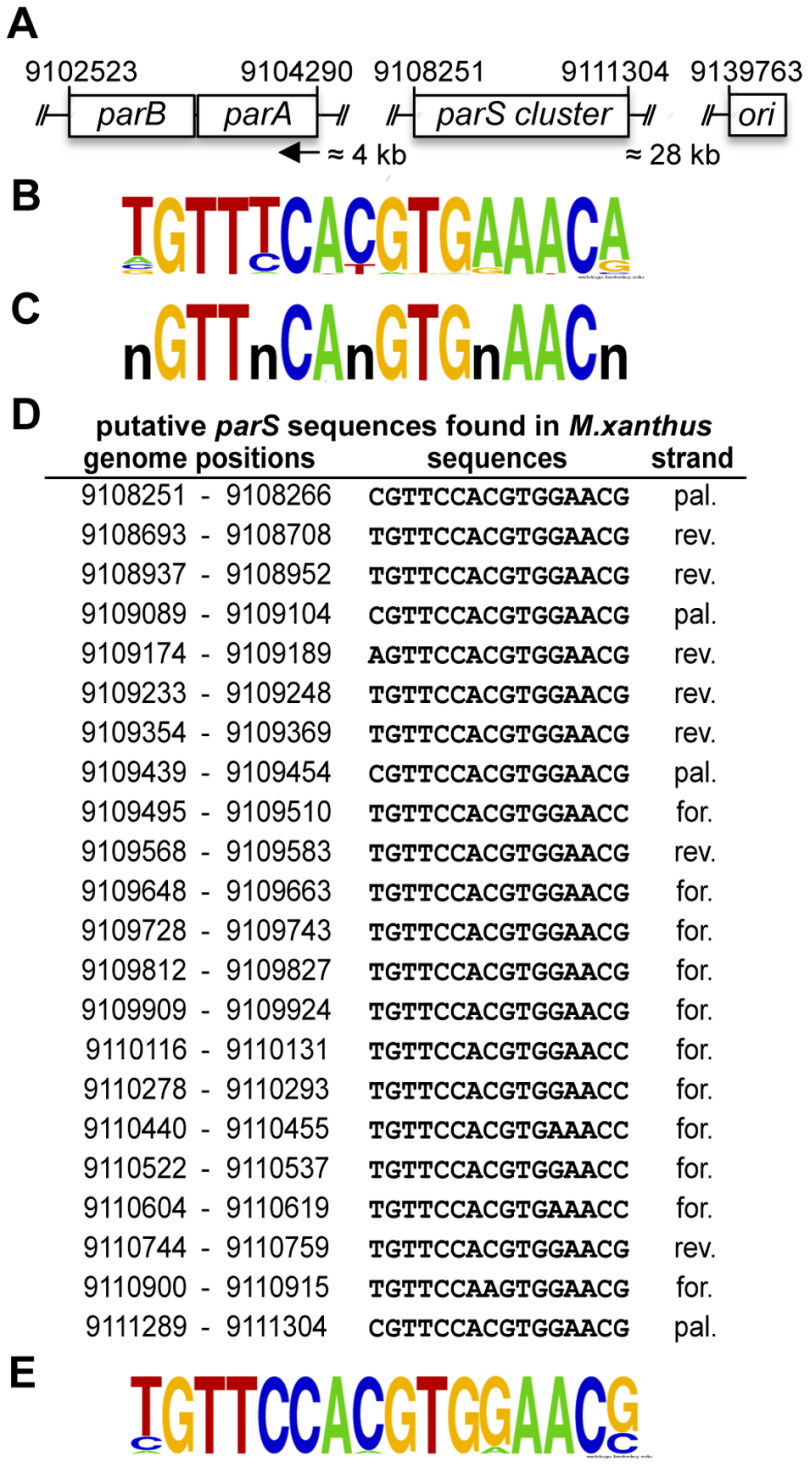
*parABS* locus in *M. xanthus*. (**A**) Genomic organization of the *parABS* locus. The arrow indicates the direction of *parA* and *parB* transcription. (**B**) WebLogo representation [60] of the consensus of 1030 *parS* sites identified from 276 prokaryotic genomes [15]. (**C**) The 16 bp sequence used to find putative *parS* sites in the *M. xanthus* genome. (**D**) Putative *parS* sequences found in *M. xanthus*, for = forward, rev = reverse, pal = palindromic (**E**) WebLogo showing the consensus of *parS* sites in *M. xanthus*.

MXAN_7477 and MXAN_7476 in the *M. xanthus* genome have been annotated as encoding *parA* and *parB*, respectively [24] (Fig. 1A). These two genes are co-transcribed as an operon together with MXAN_7475 (encoding the bactofilin BacM) and MXAN_7474 (encoding a putative lipoprotein of unknown function). Neither BacM nor MXAN_7474 is necessary for chromosome segregation or optimal cell growth under standard conditions [25]. Chromosomal *parAB* loci are usually found in the *oriC*-proximal region of bacterial chromosomes [15]. Consistent with this, the *M. xanthus parAB* locus indicated above is about 35 kb away from *oriC*. Bioinformatic analysis of 400 sequenced prokaryotic chromosomes indicated 1030 putative *parS* sites which are located, mostly, within *oriC*-proximal regions of their respective chromosomes [15]. From these *parS* sites, Livny and coworkers created a *parS* consensus matrix (Fig. 1B), and found that *M. xanthus* contains 12 putative *parS* sites, about 4.4 kb upstream from *parA*. To refine this analysis in *M. xanthus*, I decided to repeat the search for *parS* sites in the *M. xanthus* genome modifying the *parS* consensus matrix obtained previously. The search was performed using as query a putative *parS* sequence (16 pair of bases) that retains the highly conserved nucleotides, and in which the less conserved ones are varied (Fig. 1C). This search uncovered a 3 kb cluster of 22 putative *parS* sequence repeats (Fig. 1D) located about 4 kb upstream of *parA*, between genome positions 9108251 to 9111304 (Fig. 1A). In addition, this search also pointed out another *parS* site located at position 349383 to 349398, distant from the *parAB* locus. Two additional *parS* sites in the *M. xanthus* genome, near each end of the *parS* cluster, were indicated in another report using as query the 12 *parS M. xanthus* sequences previously identified and allowing for one mismatch [26]. The first sequence (position 9105392–9105407) has a “G” in position 7, and the second sequence (position 9111742–9111757) contains a “C” in position 3. Only two examples of each of these case exists among the 1030 predicted *parS* sites mentioned earlier. Positions 3 and 7 are otherwise highly conserved. The level of nucleotide conservation is important because the 1030 *parS* sites, found in 276 of the 400 sequenced strains, were identified using as a reference only the 15 *parS* sites from *Streptomyces coelicolor* and the 10 *parS* sites from *B. subtilis* that have been shown to bind ParB *in vivo* [27]–[29]. Taking the 22 putative *parS* sites described here (Fig. 1D), the *M. xanthus parS* consensus sequence can be assigned to be TGTTCCACGTGGAACG (Fig. 1E).

### *M. xanthus* ParB Binds to a Consensus *parS in vitro*

**Figure 2:**
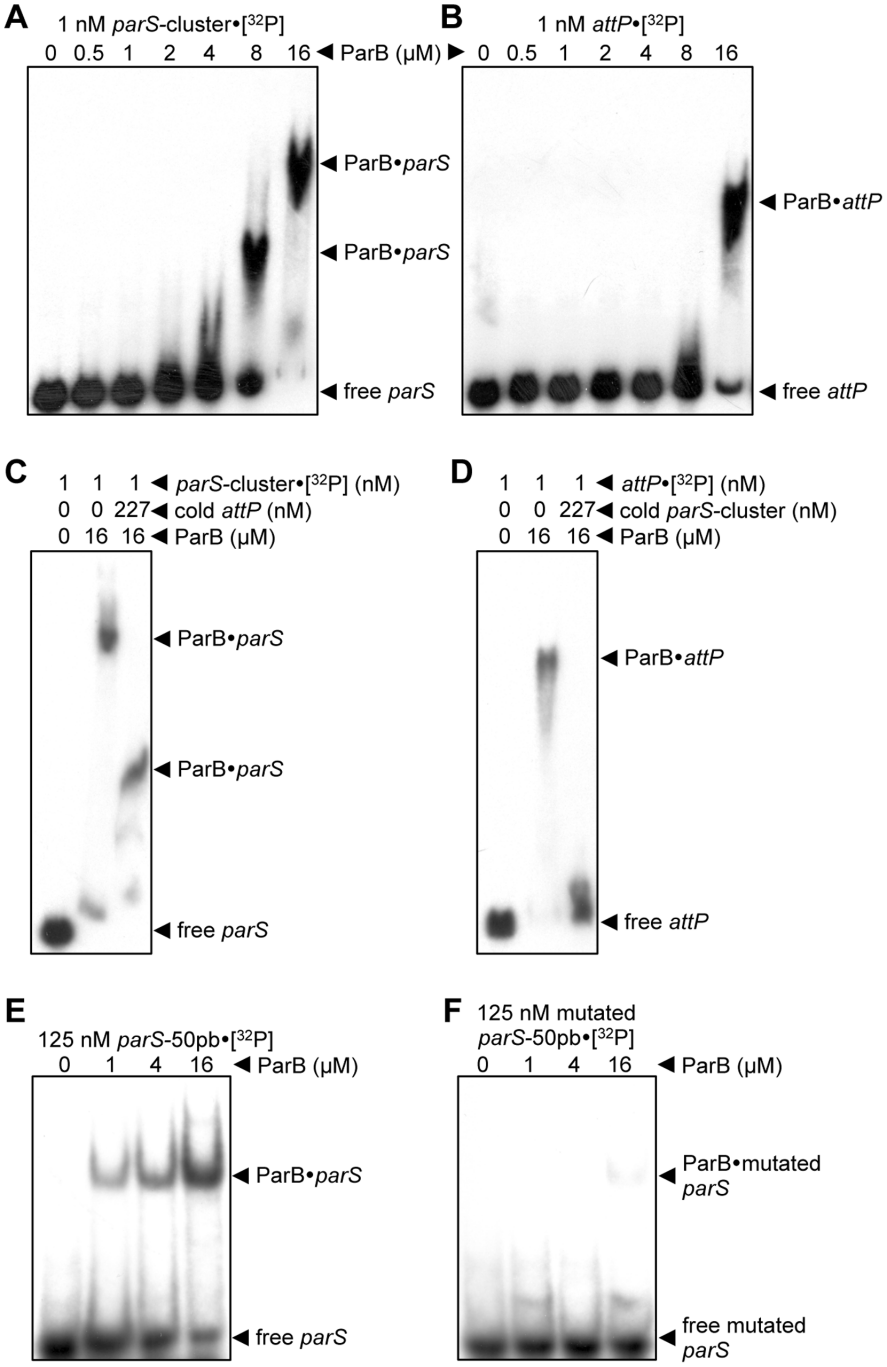
ParB binds preferentially to *parS in vitro*. (**A**) Agarose EMSA on after incubating different amounts of ParB with a ^32^P-labeled 3120 bp DNA fragment containing the *parS*-cluster, (**B**) or with a 3005 bp DNA containing the Mx8 phage *attP* sequence. (**C**) Agarose EMSA of the binding of ^32^P-labeled *parS*-cluster to ParB, or to ParB previously incubated with higher amounts of unlabeled *attP* DNA fragment, as indicated. (**D**) Agarose EMSA of the binding of ^32^P-labeled *attP* fragment to ParB, or to ParB previously incubated with higher amounts of unlabeled *parS*-cluster, as indicated. (**E**) EMSA using a 6% polyacrylamide gel after incubating a ^32^P-labeled 50 bp DNA fragment containing the *M. xanthus parS* consensus sequence with increasing amounts of ParB, as indicated (**F**) or with this ^32^P-labeled 50 bp DNA fragment containing 11 point mutations of the most conserved base pairs of *parS*.

In order to determine if *M. xanthus* ParB is able to bind to the DNA fragment containing the 22 *parS* cluster described in the above section, an agarose gel electrophoretic mobility shift assay was performed. For this, a 3.12 kb ^32^P-radiolabeled probe, corresponding to the DNA segment from positions 9108215 to 9111334 of the *M. xanthus* genome and containing the 22-repeat *parS* stretch was incubated with increasing amounts of purified ParB, and electrophoresed in a 0.7% agarose gel. Clear mobility shifts of the *parS*-cluster probe appear at ParB concentrations >2 µM (Fig. 2A). This indicates that ParB interacts with the DNA fragment containing the 22 *M. xanthus parS* sites. It can also be observed that higher amounts of ParB resulted in slower-migrating bands. It has been previously described elsewhere [29], [30], that large DNA probe fragments show multiple slower-migrating bands as ParB concentration increase, indicating that several molecules of ParB are binding per DNA fragment, generating a large nucleoprotein complex near the origin of replication. These large structures could potentially demarcate, organize or localize the origin region of the chromosome [29], [30]. To assess its preferential binding to *parS* sites, ParB binding was tested with another DNA probe similar in size and G+C content but without the *parS*-cluster (3005 bp and 65.7% G+C versus 3120 bp and 64.9% G+C). The DNA probe chosen contained the Mx8 phage *attP* site involved in phage integration into the *M. xanthus* chromosome at the *attB* site [31]. ParB could bind to the *attP* fragment but only at ParB concentrations far higher than with the *parS* probe (Fig. 2B), indicative of the significantly greater of ParB for the probe bearing the *parS*-cluster. In order to further establish the preferential binding of ParB to the sequence with the *parS*-cluster, a DNA binding competition assay was performed. Prior to the 30 minutes of ParB incubation with the labeled probe with the *parS*-cluster, ParB was incubated with unlabeled *attP* probe at more than two-hundred fold excess for 1 hour. As can be seen in Fig. 2C, ParB complexes with labeled *parS* probe appeared even with cold *attP* probe present at over a 227-fold excess. By contrast, with a similar excess of cold *parS* probe, ParB complexes with labeled *attP* probe could not be detected (Fig. 2D) confirming that ParB has a greater affinity for the probe with the *parS*-cluster.

Next, to determine if ParB binds specifically to a single *parS* site, a 50 bp DNA probe containing the *M. xanthus parS* consensus sequence TGTTCCACGTGGAACG (Fig. 1E), which spans positions 9109710 to 9109759 of the *M. xanthus* genome, was used in the gel mobility assay. ParB was found to bind to this single *parS* site (Fig. 2E) with a single retarded band observed even at the highest ParB concentration used, in contrast to the 3 kb probe with several *parS* repeats which yielded a large complex at high concentrations. However, when ParB was incubated with a similar DNA fragment but containing 11-point mutations (TaccCtgCacaAggtG) of the most conserved base pairs of the *parS* consensus sequence (Fig. 1B), only a faint band could be barely detected even at the highest ParB concentration used (Fig. 2F). These *in vitro* results are consistent with specific binding of ParB to a consensus *parS* sequence.

### ParB is Essential for Viability in *M. xanthus*

**Figure 3:**
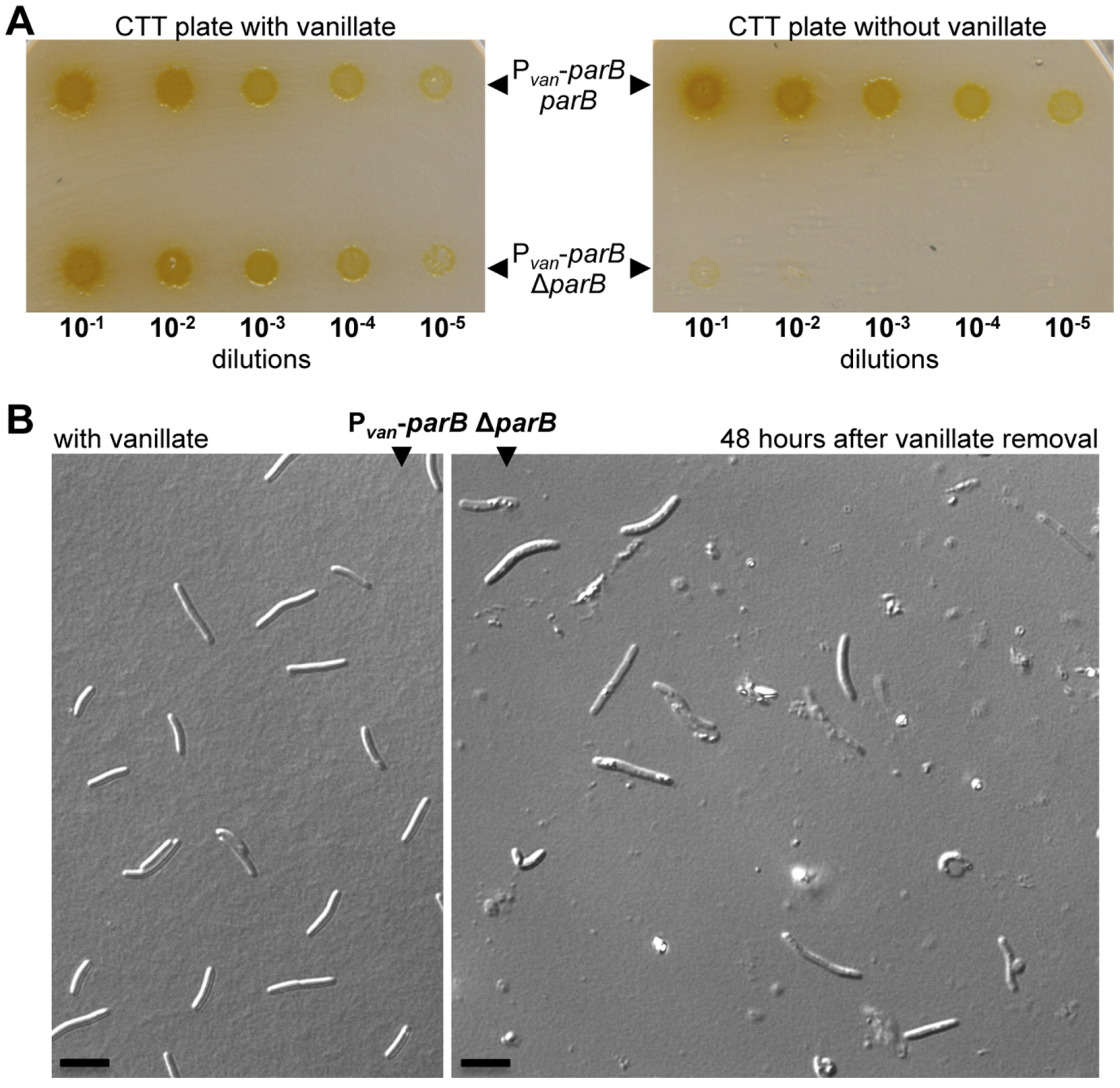
ParB is essential in *M. xanthus*. (**A**) Strains MR2461 (P*_van_*-*parB*) and MR2472 (P*_van_*-*parB* Δ*parB*) were grown in CTT media in the presence of vanillate (for *parB* expression) to an O.D._550_ of 0.8. The cultures were serially diluted, and 5 µl of each sample was spotted onto CTT plates containing vanillate (left), and no vanillate (right). (**B**) Microscope DIC images from a cell culture from the strain P*_van_*-*parB* Δ*parB* in the presence of vanillate (left image), and 48 hours after vanillate removal (right image).

ParB has been shown to be important for chromosome partitioning in numerous bacteria [14], [32]. In order to determine if *M. xanthus* ParB participates in chromosome segregation, it was necessary to create a *parB* mutant. It was not possible to delete *parB* in *M. xanthus*, suggesting that ParB is essential for viability. In fact, the endogenous *parB* gene could be deleted only if an extra copy of the gene was also present (located at the *1.38-kb* locus described in detail in reference 33). Moreover, conditional expression of *parB* by placing it under the control of a vanillate-inducible promoter, which is derepressed in the presence of vanillate [33], [34], resulted in viable cell growth only under permissive conditions when vanillate was present (Fig. 3A, left panel), whereas there was no growth under restrictive conditions without vanillate (Fig. 3A, right panel). Furthermore, restricting *parB* expression results in cells (after a 48-hour growth with no vanillate to ensure complete growth arrest) in aberrant cellular morphology when examined under a microscope, and considerable amounts of cellular debris, indicating cellular death, could be observed (Fig. 3B, right panel). Thus, these data clearly demonstrate that *parB* is essential for viability in *M. xanthus*. In most chromosomal *par* systems studied, mutations or deletions of the *par* genes did not produce lethality [14], [32], [35], a notable exception being *C. crescentus* [36]. For instance, in *Deinococcus radiodurans* and *P. aeruginosa*, the absence of ParB resulted in bacterial growth retardation [32], [37]. Additionally in *P. aeruginosa*, *parB* mutants were affected in swarming and swimming motility [32], and in *B. subtillis*, the absence of ParB ortholog Spo0J caused a sporulation defect [38]. It has been proposed that the essentiality of the *par* system in *C. crescentus* is due to a cell division defect, indicating that ParAB are required for cytokinesis [39]. Although this may also be the reason why the *par* system is essential in *M. xanthus*, the dramatic filamentous cell morphology phenotype that *C. crescentus* cells present in the absence of *parB* is not observed with *M. xanthus*.

### *M. xanthus* ParB is Involved in Chromosome Partitioning

**Figure 4:**
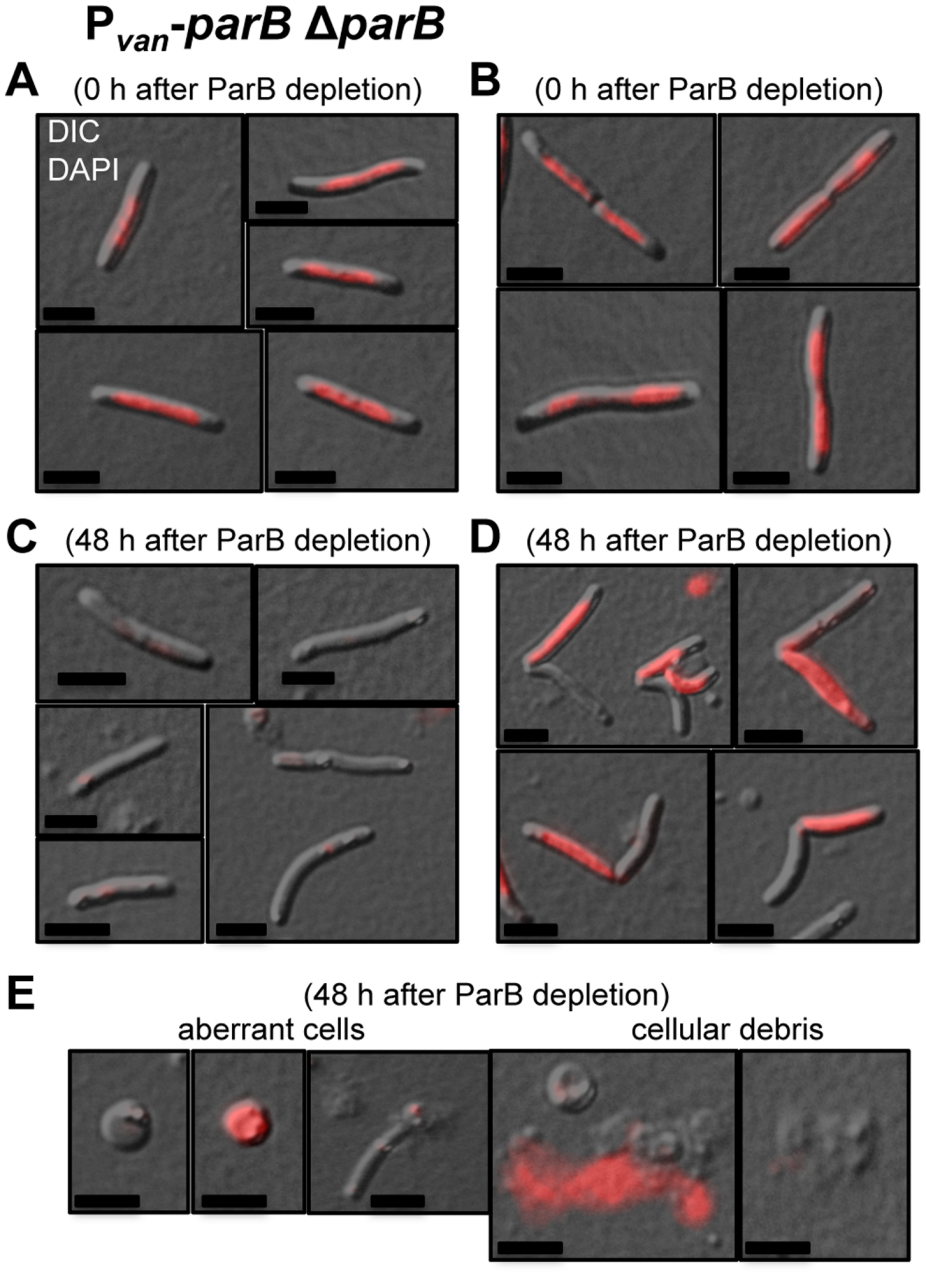
ParB is involved in chromosome partitioning in *M. xanthus*. Merged DIC and fluorescence images of cells from a P*_van_*-*parB* Δ*parB* (MR2472) culture in the presence of vanillate (permissive conditions) (**A**) and (**B**), and after 48 hours in the absence of vanillate (restrictive conditions) (**C**), (**D**) and (**E**). The cultures were stained with DAPI for viewing chromosomal DNA by fluorescence, shown in red. Black scale bars represents 5 µm. (**A**) Non-dividing cells with DNA. (**B**) Dividing cells with DNA. (**C**) Non-dividing cells without DNA. (**D**) Dividing cells with DNA only in one compartment. (**E**) Rounded cells without DNA (first from left) and with DNA (second from left), broken cell without DNA (middle), extracytoplasmic DNA (second right) and cellular debris (first right). The mean of the results from three independent experiments and the standard deviations are shown in Table 1.

Chromosomally encoded ParB or ParA proteins have been reported to have a role in chromosome partitioning in several bacteria. It is established that a lack of Par proteins or the presence of mutant forms of theses proteins causes an increase in anucleate cells [6], [28], [36]–[38], [40]–[42]. To ascertain if the absence of ParB in *M. xanthus* results in anucleate cells, a culture of *parB* conditional strain, P*_van_*-*parB* Δ*parB*, was grown in the presence of vanillate. The cell culture was washed to remove vanillate from the media and the cells were examined under the microscope after 0, 24, 36, and 48 hours. Samples were incubated with DAPI 10 minutes before being placed on the agarose pad to observe chromosomal DNA by fluorescence microscopy. In the presence of vanillate, all cells had normal rod-shape morphology and contained chromosomal DNA, with dividing cells having DNA in both compartments (Table 1, Figs. 4A and B). It should be noted that the chromosomal DNA does not occupy the entire cytoplasmic space, leaving areas proximal to the cell poles free of DNA. After 24 hours of ParB depletion, anucleate cells start to appear, together with dividing cells bearing DNA only in one compartment instead of both, although the number of cells with such anomalies is small (1% of 400 cells observed; Table 1). After 36 and 48 hours of ParB depletion, the population of anucleate cells is quite significant (between 10.1 and 21.6%, n = 310 and n = 219, respectively), and the number of dividing cells with DNA in only one compartment also increases (between 14.4 and 9.4%, n = 310 and n = 219, respectively) (Table 1, Figs. 4C and D; these counts considered only cells that conserved the typical smooth rod-shape morphology). After 48 hours of ParB depletion several rounded cells (with or without DNA), significant amounts of cellular debris, and free chromosomal DNA, presumably released from dead cells into the media, can be observed (Fig. 4E). This suggests that *M. xanthus* ParB is involved in chromosome partitioning, and its absence provokes chromosome segregation anomalies and cellular death.

**Table 1:** Presence or absence of DNA in *M. xanthus* cells depleted of ParB.

rod-shape cells	time after vanillate removal ⇒	0 h	SD	24 h	SD	36 h	SD	48 h	SD
not dividing	with DNA	91.0%	2.3	95.1%	4.1	67.9%	7.9	60.9%	5.9
	without DNA	0.0%	0.0	0.2%	0.3	10.1%	4.4	21.6%	5.9
dividing	with DNA (in 2 compartments)	9.0%	2.3	3.9%	4.1	7.6%	3.3	8.1%	1.6
	without DNA (in 1 compartment)	0.0%	0.0	0.8%	0.2	14.4%	7.4	9.4%	2.3
	total number of cells observed	272		400		310		219	

SD: standard deviation.

### Subcellular Localization of *M. xanthus* ParA

**Figure 5:**
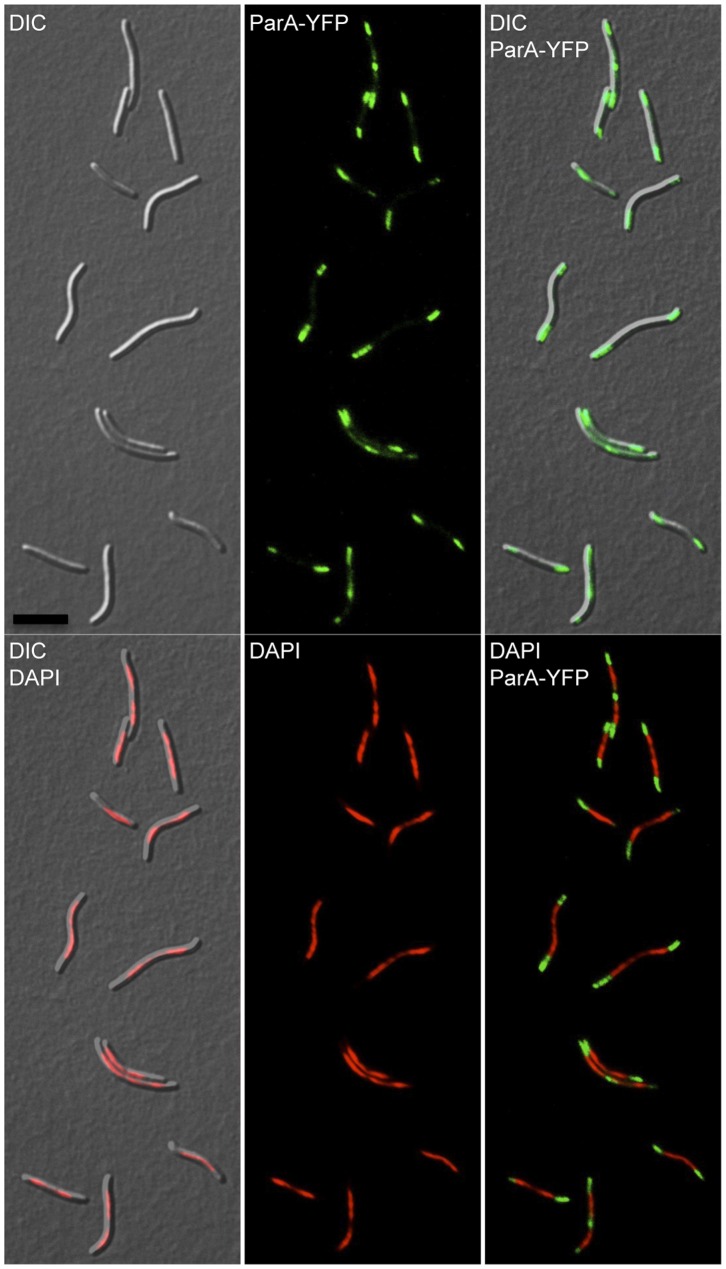
Subcellular localization of ParA in *M. xanthus*. Fluorescence microscope images of cells from the strain P*_van_*-*parA-*yfp (MR2504) grown without vanillate. DIC (top left), ParA-YFP fluorescence (top middle, in green), merged DIC with ParA-YFP fluorescence (top right), merged DIC with DAPI fluorescence (bottom left), DAPI fluorescence (bottom middle, in red), and merged DAPI (in red) with ParA-YFP (in green). Black scale bar represents 10 µm.

**Figure 6:**
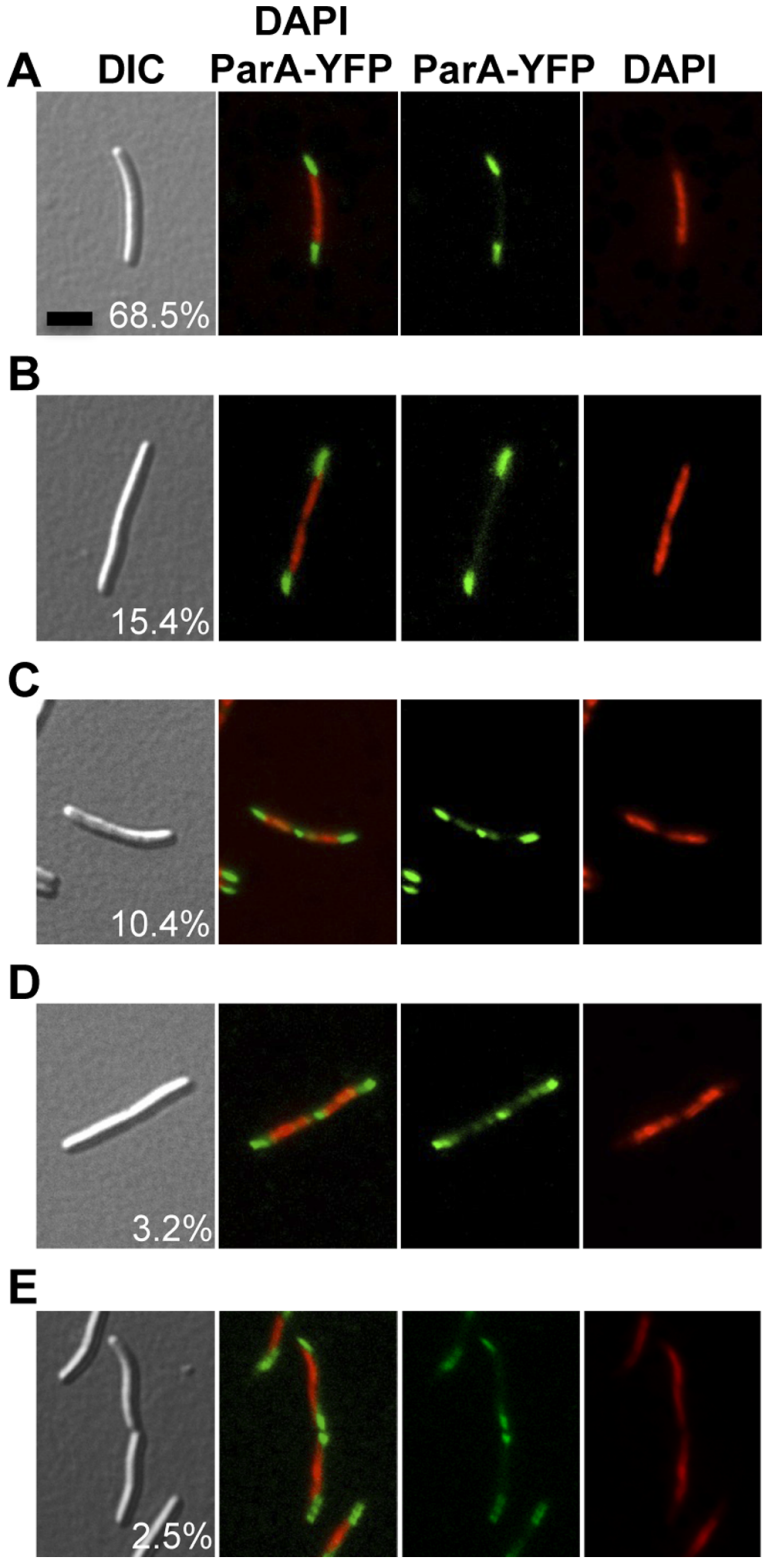
Distribution of cells according to its ParA localization. DIC, ParA-YFP (in green), and DAPI (in red) microscope fluorescence images of cells from the strain P*_van_*-*parA-yfp* (MR2504) grown without vanillate. Black scale bar represents 5 µm. A total of 615 cells from three independent experiments were examined and the mean and the standard deviation are reported. (**A**) A representative cell with two polar clusters of ParA-YFP and one chromosomal mass (68.5±10.2%). (**B**) Cell having two polar clusters of ParA-YFP and two distinct chromosomal masses (15.4±8.7%). (**C**) Representative cell with two polar clusters of ParA-YFP, two distinct chromosomal masses, and an additional cluster of ParA-YFP in the cell division plane but with no pinch in its cellular morphology (10.4±2.3%). (**D**) Same as in (**C**) but with incipient constriction along the cell division plane (3.2±0.7%). (**E**) Cells recently divided, showing two polar clusters of ParA-YFP and one chromosomal mass, in each of the two cells (2.5±1.4%).

All attempts to delete chromosomal *parA* from *M. xanthus* were unsuccessful. The chromosomal *parA* gene was only deleted when the strain harbored another copy of *parA in trans* under the control of the P*_van_* promoter, indicating that, as with *parB*, *parA* is essential for viability in *M. xanthus* (data not shown). In this case, basal levels of expression from the P*_van_* promoter without the addition of vanillate, sufficed to allow the cells to live. To determine the subcellular localization of ParA, a strain was created harboring P*_van_*-*parA-yfp*, bearing a copy of *parA* fused to *yfp*, whose expression was under the control of the inducible vanillate promoter and inserted at the *1.38-kb* locus. After growing this strain, cells were examined under the microscope and as fluorescent ParA-YFP could be observed even in the absence of vanillate, it was not included in these analyses. ParA-YFP fluorescence was found to cluster at both poles, and in some cells at the cell division plane (Fig. 5, upper panels). Merging the images obtained for ParA-YFP with that of the chromosomal DNA visualized using DAPI staining, it is clearly apparent that ParA-YFP localizes at the DNA-free regions. As mentioned in a previous section, the chromosome in *M. xanthus* does not occupy all regions of the cytoplasmic space, with DNA-free regions at the poles and, in some cells at the cell division plane (Fig. 5, bottom panels). Approximately 84% out of the 615 cells had two ParA-YFP clusters only at the two poles (Figs. 6A and B), and in the remaining cells an additional ParA-YFP cluster was observed in the cell division plane as well (Figs. 6C–E). Even after separation of the dividing cells, ParA remains localized at the newborn pole, which earlier was part of the cell division plane in the mother cell (Fig. 6E). Therefore, the polar localization of ParA appears to be a consequence of a prior location at the midcell and raises the question of how ParA is being recruited to the cell division site. In *C. crescentus*, ParA forms a cloud-like structure extending from the new pole towards the old one. The duplicated ParB-*parS* complex associates with the ParA-cloud and is pushed apart towards the new pole, by a ParB-dependent ParA-ATPase activity [16], [43]. A similar mechanism has been proposed for *V. cholerae* chromosome I [44]. It has also been suggested that the nucleoid forms a structural matrix for the assembly of a track-like structure of ParA that guides the ParB-*parS* complex movement [16], [45]. In the multigenomic aerial hyphae of *S. coelicolor*, ParA accumulates at the tip of the hyphae and it extends from the tip towards the rest of the hyphae as helical filaments providing a scaffold for a regular distribution of several ParB*-parS* complexes [46]. In *B. subtillis*, the ParA ortholog Soj localizes to the septa and as relatively faint punctuate foci within the cytoplasm [18]. In *M. xanthus*, the symmetrical ParA localization at DNA-free poles, observed in this study, seems to be incompatible with ParB-*parS* transport by ParA through a nucleoid structural matrix. This, however, should not be discarded since a faint ParA fluorescence can be detected throughout the cytoplasm even in the regions with DNA.

### The Localization of ParA is not Dependent on FtsZ

**Figure 7:**
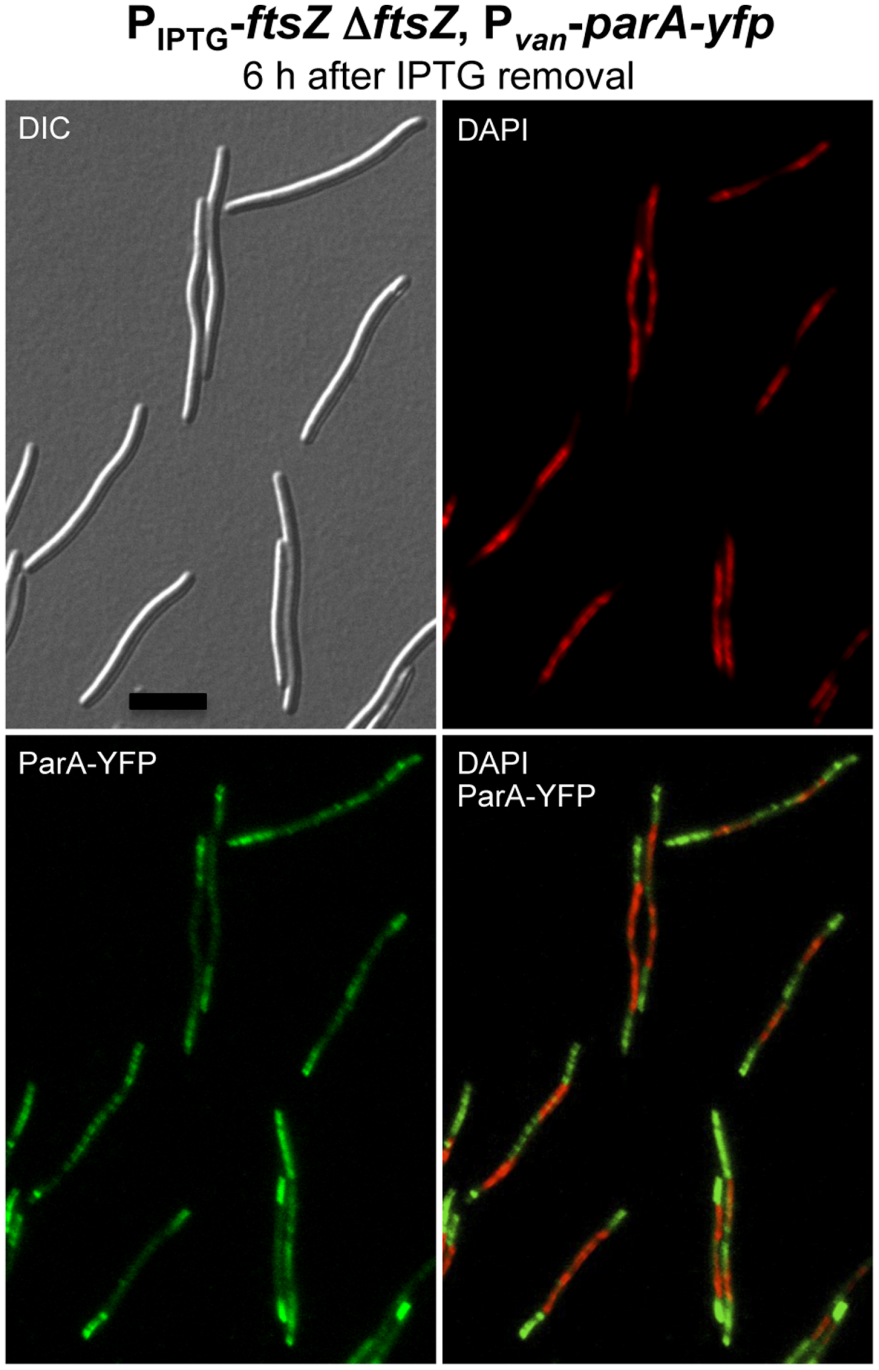
ParA localization is not dependent on FtsZ. DIC, DAPI (in red), and ParA-YFP (in green) microscope fluorescence images of cells from the strain MR2536 (P_IPTG_-*ftsZ* Δ*ftsZ*, P*_van_*-*parA-yfp*), after 6 hours of IPTG removal (FtsZ depletion). Scale bar represents 10 µm.

To obtain more insight into ParA cellular localization, this was examined in the absence of FtsZ, the bacterial tubulin homolog that forms a ring in the midcell region whose constriction culminates in cell division [47]–[49]. In *M. xanthus*, FtsZ localizes at the cell division plane in most cells, and its absence results in filamentous morphology and, eventually, cell death [33]. Whether ParA-YFP localization at the midcell depends on FtsZ, was studied by inserting a copy of P*_van_*-*parA-yfp* at the *Mxan_18–19* locus (described in detail in reference 33) in strain MR2196 to generate strain MR2536. In MR2196 the only copy of the *ftsZ* gene is under the control of the IPTG-inducible promoter, and its growth and viability depends on the presence of IPTG [33]. MR2536 was grown in the presence of IPTG, and in the absence of vanillate. Then the cells were washed repeatedly to remove IPTG and grown during 6 hours. This depletion of FtsZ produces elongated cells in which ParA-YFP continues to localize at the cell poles, and in the space between chromosomes in many cells (Fig. 7). Although in the absence of FtsZ ParA-YFP fluorescence appears to be more dispersed throughout the cytoplasm than when FtsZ is present, the overall ParA-YFP localization pattern seems to persist. Thus, the midcell localization of ParA-YFP does not appear to be correlated with that of FtsZ.

### ParB Controls ParA Localization

**Figure 8:**
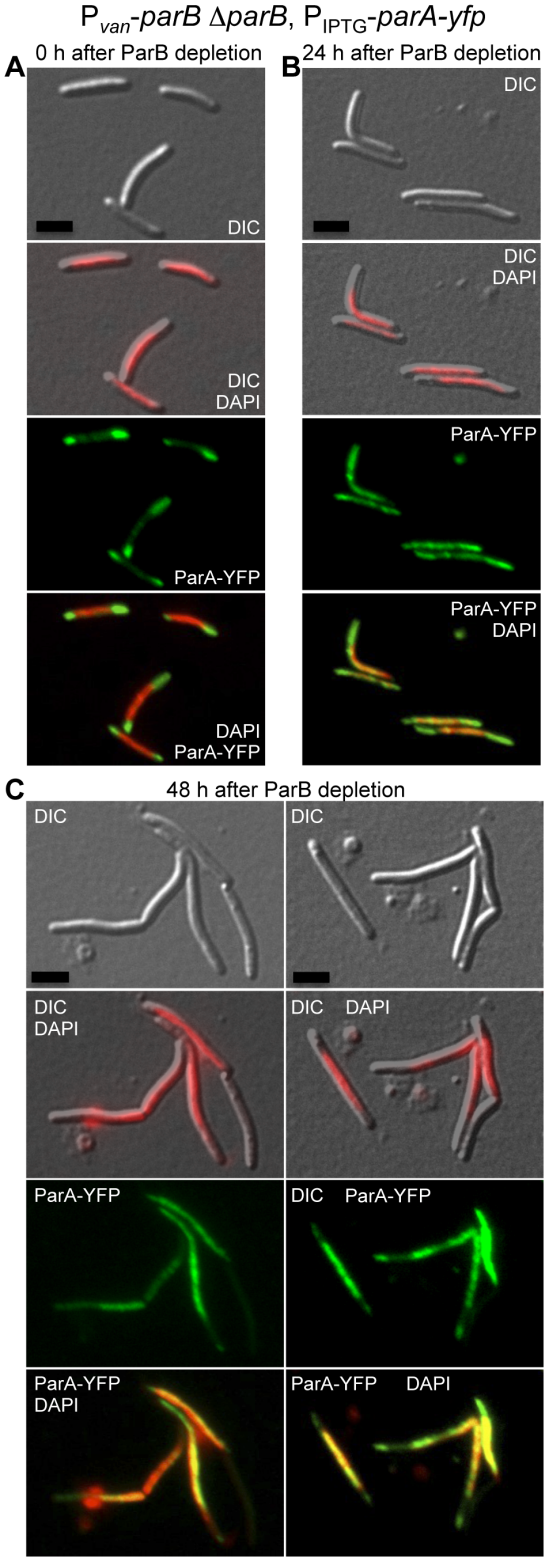
ParB inhibits ParA to localize with the nucleoid. DIC, DAPI (in red), ParA-YFP (in green), and DAPI with ParA-YFP merged (in yellow) microscope fluorescence images of cells from the strain MR2538 (P*_van_*-*parB* Δ*parB*, P_IPTG_-*parA-yfp*) grown in presence of vanillate (**A**), 24 hours (**B**), and 48 hours after vanillate removal (**C**). All images were taken 1 hour after IPTG incubation for *parA-yfp* expression. Scale bar represents 5 µm.

Since ParB has been shown to influence ParA localization in several bacteria [16], [18], [50], [51], this was tested in *M. xanthus* by examining ParA-YFP in ParB-depleted cells. For this, a copy of *parA-yfp* under the control of the IPTG promoter was inserted at the *Mxan_18–19* locus in the strain (MR2472) described earlier, which contains the only *parB* copy under the control of the vanillate-inducible promoter. The resulting strain MR2538 is thus viable in presence of vanillate in the medium. After 1 hour of IPTG-induced *parA-yfp* expression, ParA-YFP is observed as a bright signal at the DNA-free zones at the poles and, in some cells, at the cell division plane (Fig. 8A). A faint ParA-YFP fluorescence also appears throughout the rest of the cytoplasm. Therefore, localization of ParA-YFP in this strain under permissive conditions resembles that in the wild-type strain. ParA-YFP (again after 1 hour of IPTG-induction) was still observed at the poles but also overlapped with DAPI-stained DNA, 24 hours after vanillate (and hence ParB) depletion compared to when vanillate was present (Fig. 8B). Aberrant cell morphology and abnormal distribution of DNA, generating the presence of anucleate cells, were evident after 48 hours of ParB depletion, with the ParA-YFP signal (IPTG-induced for 1 hr) markedly coincident with chromosomal DNA, although it can be seen in other DNA-free zones (Fig. 8C). In anucleate cells or cellular compartments without DNA, ParA-YFP was not detected. This result indicates that, in the absence of ParB, ParA may bind to chromosomal DNA. Indeed, various studies have reported that ParA from other bacteria can bind to DNA in a nonspecific ATP-dependent manner [16], [52], [53]. Also, as observed in *M. xanthus* in this study, association of the *B. subtilis* ParA ortholog Soj to the nucleoid has been observed to occur when the ParB ortholog Spo0J is absent [18], [50], [51], and *C. crescentus* ParA-YFP heterologously expressed in *E. coli*, which lacks a Par system [15], was found to localize on the nucleoid [16].

### Subcellular Localization of ParB

**Figure 9:**
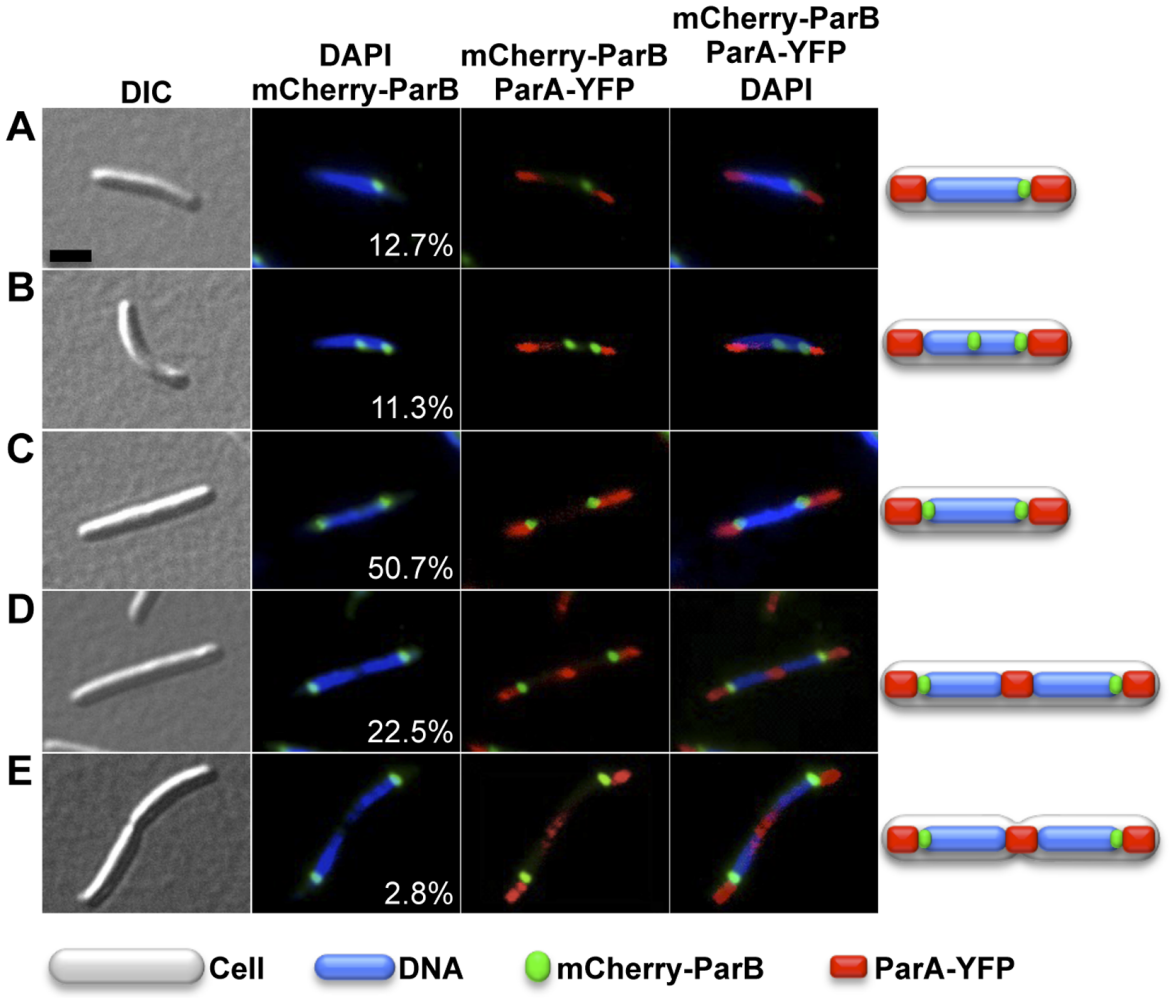
Distribution of cells according to its ParB localization. DIC, mCherry-ParB (in green), DAPI (in blue), and ParA-YFP (in red) microscope fluorescence images of cells from the strain MR2526 (P*_van_-parA-yfp,* P_IPTG_ *-mCherry-parB*) grown without vanillate, and with IPTG (1 mM) during 3 hours for *mCherry-parB* expression. Black scale bar represents 5 µm. A total of 550 cells from three independent experiments were examined and the mean and standard deviation are reported. (**A**) Cell having one chromosomal mass and a single mCherry-ParB focus at the edge of the nucleoid (12.7±1.8%). (**B**) Cell having one chromosomal mass, a mCherry-ParB focus at the edge of the nucleoid and another mCherry-ParB focus in an intermediate nucleoid position (11.3±0.3%). (**C**) Cell presenting one chromosomal mass, and two mCherry-ParB foci at both edges of a single nucleoid (50.7±3.7%). (**D**) Cell having two chromosomal masses, and two mCherry-ParB foci at the subpolar edges of both nucleoids (22.5±1.7%). (**E**) The same as in (**D**) but with some sign of cellular pinch at the cell division plane (2.8±1.5%).

To determine intracellular localization of ParB, plasmid pMR3828 encoding an mCherry-ParB fusion controlled by an IPTG-inducible promoter [33] was integrated at the *M. xanthus 1.38-kb* locus to generate strain MR2526. Additionally, strain MR2526 also has the plasmid pMR3826 with the vanillate-inducible *parA-yfp* construct integrated at the *Mxan_18–19* locus. MR2526 was grown in CTT media to exponential phase. After a 3-hours of IPTG (1 mM) induction of *mCherry-parB* expression, samples were taken and stained with DAPI for microscopy. In 12.7% of the cells observed (n = 550), a single focus of mCherry-ParB was seen just at the edge of the nucleoid (Fig. 9A). Most of the cells presented two mCherry-ParB foci at both edges of a single nucleoid (50.7% of the cells; Fig. 9C), or two foci at both subpolar edges of the two separated nucleoids (25.3% of the cells; Figs. 9D and E). The remaining cells had one focus localized at the edge of the nucleoid and another in an intermediate position (11.3% of the cells; Fig. 9B). The localization pattern of ParB in *M. xanthus* thus resembles those previously described in other bacteria, and where ParB localization was linked to its ability to bind *parS* [16], [44], [54]. Since, as shown in this study, *M. xanthus* ParB binds preferentially to a consensus *parS* sequence *in vitro* (Fig. 2E), the single mCherry-ParB focus seen in 12.7% of the cells may correspond to ParB bound to a not as yet replicated or segregated, *parS* (Fig. 9A). Then, the presence of cells having one focus at the edge of the nucleoid and other in an intermediate position (Fig. 9B) could indicate that *parS* has replicated and is being moved to the other edge of the nucleoid, resulting in cells with two ParB-*parS* clusters at both edges of the nucleoid (as seen in 50.7% of the cells). This final location of both ParB-*parS* complexes persists even after the two chromosomes have been segregated (Figs. 9D and E). Thereby, the division of the cell would provide two daughter cells with a single ParB-*parS* complex, completing the cell cycle.

Simultaneous observation of mCherry-ParB and ParA-YFP shows that ParB is in close proximity to polar ParA in 76% of the cells (Figs. 9C–E), which correspond to cells with the two ParB-*parS* complexes fully segregated. While ParB may inhibit the presence of ATP-bound ParA within the nucleoid, it may not affect ParA polymerization at the poles, where no DNA- ParB complex is present. When the two newly replicated chromosomes separate, a DNA-free space is created at the cell division plane where, presumably, the dispersed ParA could polymerize to create a new midcell ParA cluster. Therefore, the localization of ParB appears to be consistent with its role controlling ParA localization.

## Materials and Methods

### Bacterial Strains and Growth Conditions

*E. coli* strain DH5α was used for plasmid constructions and was grown at 37°C in Luria broth medium (LB) supplemented with the appropriate antibiotics. *M. xanthus* was grown at 33°C in rich Casitone-Tris (CTT) medium [55]. Media were supplemented with inducer (0.5 mM vanillate or 1 mM isopropyl β-D-thiogalactoside (IPTG)) or antibiotic (40 µg/ml kanamycin (Kan^R^), 10 µg/ml oxytetracycline (Tet^R^) for solid media, and 2,5 µg/ml oxytetracycline for liquid media), as required.

### Construction of Strains and Plasmids

*M. xanthus* strains and plasmids used in this study are listed in Table 2 and Table 3. Standard protocols and commercially available kits were used in the preparation and manipulation of chromosomal and plasmid DNA. All constructs were verified by DNA sequencing. Plasmids were introduced into *M. xanthus* by electroporation, and integration of the plasmids by homologous recombination was selected on CTT plates containing the appropriate antibiotic and/or by negative selection via a *galK* gene that confers sensitivity to galactose (Gal^S^).

**Table 2:** Relevant strains^a^.

strain	integrated plasmid(s)	relevant genotype or description	source
DK1050		*M. xanthus* wild type	[61]
DK1622		*M. xanthus* wild type	[62]
MR2461	pMR3594	*M. xanthus* DK1050 *1.38-kb*::P*_van_-parB*	This study
MR2472	pMR3594	*M. xanthus* DK1050 *1.38-kb*::P*_van_-parB*, Δ*parB*	This study
MR2504	pMR3785	*M. xanthus* DK1050 *1.38-kb*::P*_van_-parA-yfp*	This study
MR2526	pMR3826, pMR3828	*M. xanthus* DK1050 *Mxan_18–19*::P*_van_-parA-yfp*, *1.38-kb*::P_IPTG_ *-mCherry-parB*	This study
MR2536	pMR3636, pMR3826	*M. xanthus* DK1622 *1.38-kb*::P_IPTG_ *-ftsZ*, Δ*ftsZ*, *Mxan_18–19*::P*_van_-parA-yfp*	This study
MR2538	pMR3594, pMR4051	*M. xanthus* DK1050 *1.38-kb*::P*_van_-parB*, Δ*parB*, *Mxan_18–19*::P_IPTG_ *-parA-yfp*	This study

a Other strains, precursors to those listed here, are described in the text.

**Table 3:** Relevant plasmids^a^.

plasmid	relevant genotype or description	source
pMR3594	*M. xanthus 1.38-kb*::P*_van_-parB*, Tet^R^	This study
pMR3636	*M. xanthus 1.38-kb*::P_IPTG_ *-ftsZ*, Tet^R^	[33]
pMR3684	Intein_tag-*M. xanthus*-*parB*	This study
pMR3785	*M. xanthus 1.38-kb*::P*_van_-parA-yfp*, Kan^R^	This study
pMR3826	*M. xanthus Mxan_18–19*::P*_van_-parA-yfp*, Kan^R^	This study
pMR3828	*M. xanthus 1.38-kb*::P_IPTG_ *-mCherry-parB*, Tet^R^	This study
pMR4051	*M. xanthus Mxan_18–19*::P_IPTG_ *-parA-yfp*, Kan^R^	This study

a Other plasmids, precursors to those listed here, are described in the text.

*M. xanthus parB* coding sequence was PCR-amplified using genomic DNA from wild type DK1050, as DNA template, and primers 14_ParB.for (5′-gctggagtcaccatatggtgaaagcagaca-3′) and 15_ParB.rev (5′-aaaagaattcctactccttcctgagaagct-3′). This PCR product and the plasmid pMR3553, which bears the *1.38-kb* sequence for chromosome integration and the P*_van_* promoter [33], were digested with NdeI and EcoRI and ligated, obtaining the plasmid pMR3594. To generate the plasmid pMR3620 for deleting chromosomal *parB*, two PCR products were generated. The first PCR product contains about 0.92 kb of *parB* upstream sequence, and it was obtained using DK1050 genomic DNA as a template and the primers 26_UpParB-for (5′-aaaaaagcttagcagcgtggatcagcgcgc-3′) and 27_UpParB.rev (5′-aaaaatcgatcacgtcgtgactccagccag-3′). The second PCR product has around 0.94 kb of *parB* downstream sequence, and it was obtained using the primers 28_DownParB.for (5′-aaaaatcgattaggacgtggcgctccttgg-3′) and 29_DownParB.rev (5′-aaaatctagatggcacagaggaacaagtcg-3′), and DK1050 genomic DNA as a template. Both PCR products were digested with HindIII-ClaI and ClaI-XbaI, respectively, and cloned into HindIII-XbaI-digested pBJ114 plasmid, which contains the *galK* gene [56]. To generate plasmid pMR3785, the *parA* gene was PCR-amplified to be translationally fused to *yfp*, using the primers 56_ParA.for (5′-aaaaaacatatggtgcactgcatcacgcgc-3′) and 57_ParA.rev (5′-aaagaattcccagccacgcgcctgcgagggct-3′), and DK1050 genomic DNA as a template. After NdeI-EcoRI digestion, *parA* from this PCR product was cloned into a NdeI-EcoRI-digested pMR3653 plasmid [33], exchanging *ftsZ* gene for *parA.* The plasmid pMR3826 was made cloning the *parA-yfp* sequence, by digesting pMR3785 with NdeI and NheI, into the plasmid pMR3690 [33] previously digested with the same restriction enzymes. To make plasmid pMR3828, the *M. xanthus parB* coding sequence was PCR-amplified using *M. xanthus* DK1050 genomic DNA as a template and the primers 18_ParB.for (5′-aaagaattccgtggtgaaagcagacatgca-3′) and 15_ParB.rev (5′-aaaagaattcctactccttcctgagaagct-3′), digested by EcoRI and cloned into the EcoRI-digested vector pVCHYN-2 [34], resulting in the plasmid pMR3733 encoding an *mCherry-parB* fusion. Then, *mCherry-parB* fragment was amplified by PCR using plasmid pMR3733 as DNA template, and the primers 49_mCherry.for (5′-aaaatctagaatggtgagcaagggcgagga-3′) and 42_ParB.rev (5′-aaaatctagactactccttcctgagaagct-3′). This PCR product was digested by XbaI and cloned into XbaI-digested plasmid pMR3487 [33], resulting in the plasmid pMR3828 which has the *mCherry-parB* fusion under the control of a IPTG-inducible promoter, and the *1.38-kb* sequence for chromosomal integration. Plasmid pMR3684 used for ParB purification was obtained isolating the *parB* coding sequence fragment after the digestion of plasmid pMR3594 by NdeI and EcoRI, and cloning into these sites in pTYB12 (New England Biolabs). In order to create plasmid pMR4051, it was necessary to generate two precursor plasmids. First, a DNA fragment of 1.861 kb containing the IPTG inducible promoter, a multicloning site, and the *lacI* gene repressor was obtained digesting pMR3487 [33] with PstI and NdeI. This fragment was cloned into a PstI-NdeI-digested pMR2700 plasmid [57], generating the plasmid pMR4046. The plasmid pMR4046 was digested with HindIII, releasing the *M. xanthus 1.38-kb* sequence for chromosomal integration, and ligated with *M. xanthus Mxan_18–19* sequence, used for a chromosomal integration in a previous work [33], obtaining the second precursor plasmid pMR4048. The 1.319 kb *Mxan_18–19* sequence was isolated after HindIII digestion of plasmid pMR3691 [33]. Finally, a PCR-amplified *parA-yfp* sequence, obtained using pMR3785 as DNA template and the primers 97_parA.for (5′-aaaaaatctagaatggtgcactgcatcacgcg-3′) and 98_yfp.rev (5′-aaaaaaggtaccttacttgtacagctcgtcca-3′), was digested with KpnI and XbaI, and cloned into a KpnI-XbaI-digested pMR4048, producing the plasmid pMR4051.

To generate the *M. xanthus parB* conditional mutant strain, MR2472, the wild type DK1050 strain was electroporated with plasmid pMR3595, obtaining the strain MR2461. This strain contains the P*_van_-parB* sequence integrated at the *M. xanthus 1.38-kb* locus. Then, MR2461 was electroporated with plasmid pMR3620, which contains sequences upstream and downstream of *parB* in the genome to generate a *parB* deletion and the *galK* gene, creating strain MR2462. MR2462 was grown for several generations with 0.5 mM of vanillate and no Kan and plated on CTT plates supplemented with 2% galactose and 0.5 mM of vanillate to select for the loss of the Gal^S^ marker. This evicts vector DNA bearing either wild type *parB* or the Δ*parB* allele by intramolecular recombination events. Gal^R^ Kan^S^ colonies were diagnosed by PCR to isolate a strain harboring the inducible P*_van_-parB* construct and the Δ*parB* allele (MR2472). The strain MR2504 was obtained electroporating plasmid pMR3785 into the wild type DK1050 strain. The strain MR2526 was obtained electroporating the strain MR2520 with plasmid pMR3826, and MR2520 by electroporating DK1050 with plasmid pMR3828. The strain MR2536 was generated by electroporating MR2916, the strain that conditionally expresses *ftsZ* from an IPTG-inducible promoter [33], with plasmid pMR3826 in presence of 1 mM IPTG. The strain MR2538 was obtained by electroporating the *parB* conditional mutant strain MR2472 with plasmid pMR4051, in presence of 0.5 mM vanillate.

### ParB Expression and Purification

To overexpress intein-tagged *M. xanthus* ParB, 10 ml starter culture of freshly transformed *E. coli* BL21(DE3) containing plasmid pMR3684 was grown at 37°C in LB medium with 100 µg/ml of ampicillin (Amp) to an OD_600_ of 0.6. It was added to 1 l of fresh LB/Amp, grown at 37°C to an OD_600_ of 0.55, and after 30 min incubation at 18°C, overexpression of intein-tagged ParB was induced overnight at 18°C with 1 mM IPTG. After overnight induction with IPTG, cells were harvested by centrifugation (15 min at 5000×*g*) and the pellet was stored at –70°C until further use. Intein-tagged ParB was purified using chitin resin and the intein was removed by on-column intramolecular cleavage in the presence of 50 mM dithiothreitol using the IMPACT kit protocols (New England Biolabs). The cleaved protein was passed through a small amount of chitin resin a second time to remove residual intein and dialyzed extensively against 25 mM Tris pH 8, 50 mM NaCl, 5 mM MgCl_2_, 0.1 mM EDTA, 10% glycerol and 2 mM β-mercaptoethanol.

### Mobility Shift Assays

Electrophoretic mobility shift assays (EMSA) in agarose gels: The 3120-bp DNA probe containing the 22-repeat *parS-cluster* was obtained by PCR using primers 58_parS.for (5′-ccgttcgctttcgtgacgggtccaggttcc-3′) and 59_parS.rev (5′-agtaacgcagcgtcagcaccacttcgacgt-3′) ^32^P end-labeled with [γ-^32^P]ATP and T4 polynucleotide kinase, and DK1050 genomic DNA as a template. For the 3005-bp *attP probe*, the primers used were 78_attP.for (5′-aaaaaaaagcttggggatggagccagacgg-3′) and 79_attP.rev (5′-aaaaaaaagcttgggatgcggtggaccatg-3′), and pMAT4 [58] as DNA template. 15 µl samples with DNA at 1 nM, was incubated with *M. xanthus* ParB protein for 30 minutes at 30°C in binding buffer (40 mM Na-phosphate pH 8, 20 mM NaCl, 7% glycerol, 20 µg/ml BSA, and 100 µg/ml sheared salmon sperm DNA), and loaded onto an 0.7% agarose gel and run at 100 V at 4°C in 0.5× TBE buffer (45 mM Tris base, 45 mM boric acid, 1 mM EDTA). Gels were dried and analyzed by autoradiography. In competitive binding assays, 227 nM of unlabeled DNA probe was incubated with ParB protein for 1 hour at 30°C, before the inclusion of the 1 nM ^32^P-labeled DNA sample.

EMSA in polyacrylamide gels: A 50 bp DNA duplex that contains a *parS* site was generated diluting oligonucleotides 60_parS.hib (5′-tgctcgagtcatccttcgttccacgtggaacacggaggccatgagtgagt-3′) and 61_parS.hib for parS (5′- actcactcatggcctccgtgttccacgtggaacgaaggatgactcgagca-3′) to a final concentration of 5 µM each. A 50 bp DNA duplex that contains a mutated *parS* site was generated diluting oligonucleotides 89_parS.hib (5′- tgctcgagtcatccttcaccttgtgcagggtacggaggccatgagtgagt-3′) and 90_parS.hib (5′- actcactcatggcctccgtaccctgcacaaggtgaaggatgactcgagca-3′) to a final concentration of 5 µM each. Each mixture was heated to 95°C for 10 min and slowly cooled to room temperature, and then ^32^P end-labeled with [γ-^32^P]ATP and T4 polynucleotide kinase. Labeled DNA sample was incubated for 1 hour at 30°C with *M. xanthus* ParB protein in binding buffer, and 15 µl samples were loaded on a 6% polyacrylamide gels (37.5∶1 acrylamide:bis-acrylamide), and run at 150 V at 4°C. Gels were dried and analyzed by autoradiography.

### Microscopy

Samples (100 µl) of *M. xanthus* cultures taken at an optical density of 0.1 at 550 nm were incubated, when appropriate, with the fluorescence dye 4′-6-diamino-2-phenylindole (DAPI) to achieve a final concentration of 2 ng/µl for 10 minutes. A 1 µl drop of this mixture was immobilized on 1% agarose (Pronadisa) slices prepared in TPM medium (10 mM Tris-hydrochloride pH 7.6, 1 mM KH_2_PO_4_-K_2_HPO_4_ pH 7.6, and 8 mM MgSO_4_). Cells were visualized with Nikon Eclipse 80i microscope equipped with a Nikon Plan Apo VC 100×/1.4 differential interference contrast (DIC) objective and a Hamamatsu ORCA-AG charge-coupled-device camera. Images were processed with Metamorph version 4.5 (Universal Imaging Group) and Photoshop CS3 10.0 (Adobe Systems). Each reported image is representative and was verified in at least three separate experiments.

## Addendum in Proof

While this paper was under review, similar findings were reported by Harms et al. (2013) [59], who also showed that ParB and ParA are essential proteins, examined their subcellular localization patterns, and confirmed the *in vitro* binding of ParB to a consensus *parS* sequence and ParB participation in chromosome partitioning. The present work suggests that, in addition, ParB helps in correct chromosome segregation by inhibiting the nonspecific interaction between ParA and DNA and thereby prevents ParA colocalization with chromosomal DNA. It is also shown here that the polar and mid-cell localization pattern of ParA does not depend on the presence of FtsZ, the critical element for bacterial cell division.
